# A modular library for fast prototyping of solution-state nuclear magnetic resonance experiments

**DOI:** 10.5194/mr-5-51-2024

**Published:** 2024-04-23

**Authors:** Michał Górka, Wiktor Koźmiński

**Affiliations:** Biological and Chemical Research Centre, Faculty of Chemistry, University of Warsaw, Żwirki i Wigury 101, 02-089 Warsaw, Poland

## Abstract

We present a framework library (Modular Elements, ME) for the development of pulse sequences for Bruker spectrometers. It implements a two-level abstraction approach – the lower level comprises basic functional elements of pulse sequences and the higher one often-reused blocks comprising several evolution periods. The low-level abstractions reduce code duplication between variants of experiments such as hard-pulse and selective variants of individual NMR experiments. The high-level modules enable further reuse of pulse program code and aid in the construction of complex experiments. We show the library's functionality by presenting pulse programs that can be switched between standard and TROSY variants as well as hard and shaped pulses and that can seamlessly incorporate real-time homodecoupling. Adaptability is further demonstrated in a configurable 4D NOESY program.

## 1 Introduction

NMR is an extraordinarily powerful and adaptable spectroscopic method, with just the solution-state variant being capable of discerning the structure and dynamics of molecules ranging in size from simple organic compounds to large protein complexes such as a proteasome (Sprangers and Kay, 2007). The variety of experimental objects and the great number of parameters that can be measured have led to the proliferation of not only general experimental schemes, such as an ${}^{1}$ H, ${}^{15}$ N HSQC (Bodenhausen and Ruben, 1980) or a HNCO (Kay et al., 1990b; Ikura et al., 1990), but also their variants and thus the pulse sequences, implementing them as computer code. As an example, for the often-used HNCO experiment, the non-exhaustive list of meaningful implementation choices is as follows: the experiment can use hard pulses or avoid saturating water using selective pulses (Schanda et al., 2006); the final transfer element can be a simple spin echo (Palmer et al., 1991), a set of three echoes implementing a sensitivity-enhanced transfer or one of many TROSY variants (Salzmann et al., 1999b; Nietlispach, 2005), with possible optimisations (Salzmann et al., 1999a; Schulte-Herbrüggen and Sørensen, 2000); and radiation damping can be suppressed with bipolar gradients (Sklenar, 1995). Even without implementing all specialised experiment variants, the standard library supplied with the TopSpin software (Bruker) contains over 1000 pulse programs.

A common problem with pulse sequences, especially in biological NMR, is thus the requirement to code multiple variants of a given sequence. If this is done in separate files (as in the TopSpin built-in library), it results in a lot of code repetition, and, if done using conditional statements, it can substantially complicate the structure of the file, making troubleshooting harder. Similarly, many pulse sequences share large amounts of code, often with no or minimal changes. Because this repeated code is scattered across different sequences and variants of experiments, adding new variants (using different soft pulses or adding homodecoupling) requires the same modification to be applied across a large part of the whole pulse sequence library, which is tedious and error-prone. It is possible to implement such a library using a standard systems programming language like C or Python, but we decided to use the native programming language of the spectrometer system, since any user writing a pulse sequence needs to be familiar with it, and requiring knowledge of a separate programming language and its tooling would be an unnecessary hurdle to adoption. Here we show that, by abstracting certain functionalities using the somewhat limited macro and define statements built into the TopSpin software, the problems described above can still be avoided and the code can be made more readable and easier to modify. Here, we present the Modular Elements (ME) library for Bruker spectrometers. Although the library is specific to a particular hardware vendor, the modular approach it implements is more general and can be implemented on other instruments. A previous implementation of a modular library for pulse program implementation (NMR blocks) can be found in Zawadzka-Kazimierczuk (2012) for Varian/Agilent spectrometers, where evolution periods such as INEPT or COS-INEPT were abstracted as C functions. Alternative approaches to a modular library include domain-specific pulse program generators, like GENESIS (Yong et al., 2022) for NOAH supersequences. Specialised libraries combining custom pulse programs and various tools (Favier and Brutscher, 2019; Vallet et al., 2020; Lukavsky and Puglisi, 2001) are suitable for routine use but have limited applicability in the prototyping of new sequences.

## 2 General approach to pulse sequence modularisation

We categorise the library's functionality as low and high level. Low-level functionality encompasses the creation of variables and functions (technically functional macros), abstracting the basic building blocks of pulse sequences like pulses, gradients and delays. A pulse function can expand to a 90° proton hard pulse or an HN selective excitation pulse, depending on the global settings. A gradient function can expand to “no operation” in standard HSQC or a selection gradient in the gradient-selected HSQC variant, with its corresponding delay variable containing a zero or correspondingly non-zero length of time. Decoupling functions for protons and deuterium can likewise be enabled and disabled depending on whether a TROSY variant is desired and whether the sample is deuterated. This functionality simplifies the writing of pulse sequences implementing multiple variants of a given NMR experiment and gives its user the ability to easily test and compare the effectiveness of the variants for a given sample, and the commonisation of parameters across variants enables faster optimisation.

High-level functionality is implemented as modules that are included in the pulse sequences and that can be classified as general modules and specific modules. General modules implement elements common to almost all pulse sequences. The functionally most significant ones are the preparation and acquisition modules. The preparation module gives the user the option of turning on functionality such as solvent presaturation or a combination of N/C pulses and pulse field gradients for spoiling the residual magnetisation on those nuclei. The acquisition module enables switching between standard or homodecoupled data acquisition. The specific modules are abstract blocks of pulse sequence elements that appear in many pulse sequences in an almost identical form. The two main types of specific modules are proximal and distal modules, abstracting the functionality of blocks including and following first excitation (distal) and right before acquisition (proximal). Despite a large variety of possible implementations, the proximal and distal fragments differentiate between variants of a pulse sequence (e.g. a standard hard-pulse HNCO, selective or BEST HNCO, or a hard-pulse and BEST TROSY-HNCO; Solyom et al., 2013), and the actual code is usually repeatable across different sequences. HNCO, HNCACO and HNCOCA (Yang and Kay, 1999) have very similar proximal and distal parts; HN(CA)CONH and HabCabCONH (Kazimierczuk et al., 2010) have different proximal blocks, but the distal block is still very similar for all the sequences listed. With the use of the low-level functionality described above, a single proximal module can abstract the initial two transfer periods (first with transverse H magnetisation and second with transverse N/C magnetisation), with the choice of N/C nucleus and the choice of evolved J coupling (CO in HNCO) made using a define directive in the main pulse sequence. NOESY experiments are particularly susceptible to modularisation, with the NOE transfer period naturally splitting them into proximal and distal blocks. Standard 2D experiments of the HSQC, TROSY and HMQC types have thus been implemented as proximal modules that can be used on their own as 2D experiments or included in a 3D or 4D NOESY (Kay et al., 1990a) with the chosen distal modules, which can themselves be modified 2D experiments or simpler blocks.

## 3 Library implementation

Description of implementation details and design choices requires a quick recapitulation of TopSpin pulse programs' language specifics. TopSpin has two types of variables: user-adjustable numbered variables (*d1*–*d63* for delays, *cnst1*–*cnst63* for floating point constants and similar for integer constants (“loopcounters”) *lN*, pulse lengths *pN*, etc.) and named variables (pulses, delays and loopcounters only, as well as lists of various kinds), which can only be manipulated within a pulse program. Some less-documented observations on the limitations of named variables are compiled in the library documentation (Górka and Koźmiński, 2024a). TopSpin implements limited functionality for defining text-substitution macros (the “-traditional” mode of the GNU C preprocessor cpp; Stallman and GCC Developer Community, 2012), which can be used everywhere outside a “relation” (variable value calculations using a subset of C syntax) due to their implementation as text in quotes (treated as string literals by cpp and ignored for macro expansion), though this limitation can be overcome (see the file “notes on TopSpin.txt” in the ME library). The user can provide custom option choices to a pulse program using the ZGOPNTS variable to define appropriate macros.

### 3.1 Low-level modularisation

#### 3.1.1 Variables

With no user-adjustable named variables, two approaches to making them consistent across different pulse programs are possible – indirection through named variables or introduction of a convention attaching constant meanings to numbered variables. Due to the limited number and type of named variables, we predominantly use the latter option (with sets of variables described in files such as delays.incl, pulse.incl, etc.) with some focused use of indirection – for example, proximal-type modules use *timeHX* and *timeXY* for J coupling evolution times between the H, X and Y nuclei. Default values for all such variables can by set using the me.set_parameters.py TopSpin program. For variables that do not ordinarily have calculations performed on them (pulse phases *phN*, gradient programs *gpN*), we implemented full indirection, where the user can use phFree1 or phFree3 without worrying about which phN variables are used by other parts of a pulse program.

#### 3.1.2 Pulses

The most important low-level abstractions are pulse functions. They are implemented using function-like macros of cpp and have the general form nucleus_type(phase), where “nucleus” can be a general specifier like H/C/N or more specific like HN/HC/CA/CO and type is classified based on the desired functionality, with the main ones being excitation (for the excitation of longitudinal magnetisation), flipback (acting on transverse magnetisation), refocusing and inversion (inverting longitudinal magnetisation) and excitation_UR and flipback_UR (implementing universal rotations). The pulse macros will have a different replacement text based on global settings (usually ZGOPTNS). A proton pulse “H_excitation(ph)” will be replaced with a hard pulse “p1 ph pl1” by default, but a “-DH_SHAPED” option will instead be replaced with “p54:sp54 ph” for a selective soft pulse and the associated named variable “pH_excitation” will be set to have the same value as *p1* or *p54*.

Pulse programs should account for the effective evolution time during pulses (which can be as much as 1 ms for longer selective pulses) to give correctly phased spectra and optimal J -coupling evolution times. This library only accounts for linear phase slopes using the modelling method described in Lescop et al. (2010), i.e. treating a pulse as a sequence (delay, ideal pulse, delay), which accounts for the phase slope of many commonly used pulses and which can be explicitly optimised during pulse design (Gershenzon et al., 2008; Asami et al., 2018). This phase slope is compensated for using variables such as *eH_excitation*, which for the hard pulse above would be set to $\frac{2p1}{\pi }$ . We assume that the flipback and flipback_UR pulses act as if they were time-reversed excitation pulses, and so the effective evolution time for a flipback pulse acting on transverse magnetisation is also *eH_excitation*. A H_excitation_UR pulse of phase x will give effective times of *eH_excitation* for z magnetisation, *eH_flipback* for y magnetisation and *eH_excitation* + *eH_flipback* for x magnetisation. By compensating for delays using the above-mentioned variables, the whole sequence can be switched from a hard-pulse implementation to a shaped-pulse version to account for field inhomogeneity or to perform band-selective excitation.

#### 3.1.3 Code blocks

There are many small blocks of code that can be included or excluded in a pulse program based on a sequence variant. To limit the number of conditional statements in the main pulse program, many are defined as macros that will expand to pulse program code based on options. For example, “H2O_FLIPBACK(ph2)” will be replaced with “(11:sp1 ph2):f1” in a pulse sequence with water flipback and with whitespace when using selective pulses. Similarly, DECOUPLE_H_ON and DECOUPLE_H_OFF macros will turn on proton decoupling in a standard HNCO experiment but will have no effect in TROSY-HNCO.

### 3.2 High-level modularisation

TopSpin pulse programs follow a defined sequential structure that complicates the implementation of high-level modules as individual files and that, in general, consists of

1. configuration and compile-time calculations,
2. a “zd” or “ze” statement,
3. a pulse program body (pulses and delays) and real-time calculations,
4. a signal acquisition block,
5. loop statements for scans of a free induction decay (FID) and points of a multi-dimensional experiment, and
6. phase program definitions.

#### 3.2.1 General modules

The general modules fit into this sequential structure as follows.

1a. Configuration and compile-time calculations
1b. init. incl
1c. Configuration and compile-time calculations continued
2. A “zd” or “ze” statement
3a. Real-time calculations
3b. start.incl
3c. A pulse program body (pulses and delays) and real-time calculations
4. end.incl
5. Loop statements for scans of a FID and points of a multi-dimensional experiment
6a. phasecycles.incl
6b. Phase program definitions

The general modules have numerous conditional statements and imports evaluating the option provided in point (1) above and using the built-in ZGOPTNS variable to interact with the specific modules (this is covered below). The init.incl module provides the library's core functionality by defining macros for functions and variable descriptions; start.incl executes the relaxation delay (with possible solvent presaturation) and optional operations, such as crushing residual C or N magnetisation (gradient pulse after an excitation pulse) or inverting N magnetisation before the relaxation delay in BEST-TROSY. For non-protein experiments, an acceleration by sharing adjacent polarization (ASAP) (Kupče and Freeman, 2007) period would be added here, but the relevant code is experimental and provided in a commented-out form due to the method's potential to damage probe heads. The end.incl module handles acquisition with the option for real-time homodecoupling – here provided with the ${}^{13}$ C-GBIRD ${}^{r,X}$ (Garbow et al., 1982; Haller et al., 2022) and BASHD (Brüschweiler et al., 1988; Krishnamurthy, 1997) types.

#### 3.2.2 Specific modules

In contrast to the general modules, specific modules implement a specific form of a proximal or distal block and serve to localise the relevant code in a single file. The biggest hurdle to writing self-contained modules for TopSpin is the sequential pulse program structure necessitating the separation of related code segments in the post-preprocessing file. To mitigate this problem, each module is entirely enclosed in a conditional statement with alternative conditions (an if…elif…else structure), and including the file once will only insert a selected part of the module into a file. Since the four general modules already perform the sequential separation of code, each of them sets the appropriate conditions (defines a macro) and imports distal_2D.incl and proximal_2D.incl, which themselves import the selected specific modules at each of the four positions in the pulse program. Thus, the initialisation-phase statements (variable declarations, some calculations, macro definitions) are included in init.incl, and runtime calculations of both types of modules are included through start.incl together with the main body (pulses and delay statements) of the distal block. Similarly, the main body of the proximal module is included through end.incl before the latter's acquisition portion. Phase cycles of both modules are inserted into a pulse program file through phasecycles.incl with some basic logic, allowing for coordination of the cycles between them if two modules are used.

For triple-resonance experiments (in the implementation limited to amide protons, but it should be possible to extend them to aliphatic and aromatic groups), the proximal module hx.incl and the distal module hx.incl provide the ability to compartmentalise the relatively standard blocks for both out-and-back- and straight-through-type experiments, and a more detailed description in the context of a HNCO experiment is provided below. Although sub-optimal under some circumstances, the library provides default two-step phase cycles for each of the modules, leaving the implementation of eight-step and longer cycles for the central part of the program. A more detailed description of the individual modules is provided in the library documentation. In the Supplement we provide a detailed step-by-step description of the proximal HSQC module and the way it is used in the 2D experiment pulse program.

A specific module separate from the proximal and distal types can also be based on the same structure and manually included either in the pulse program after each general module or in a specific module itself – the se.incl module implements the sensitivity-enhanced COS-INEPT and TROSY transfers and is imported into both the hsqc_se.incl and hx.incl modules.

## 4 Application examples

### 4.1 HNCO

**Figure 1:**
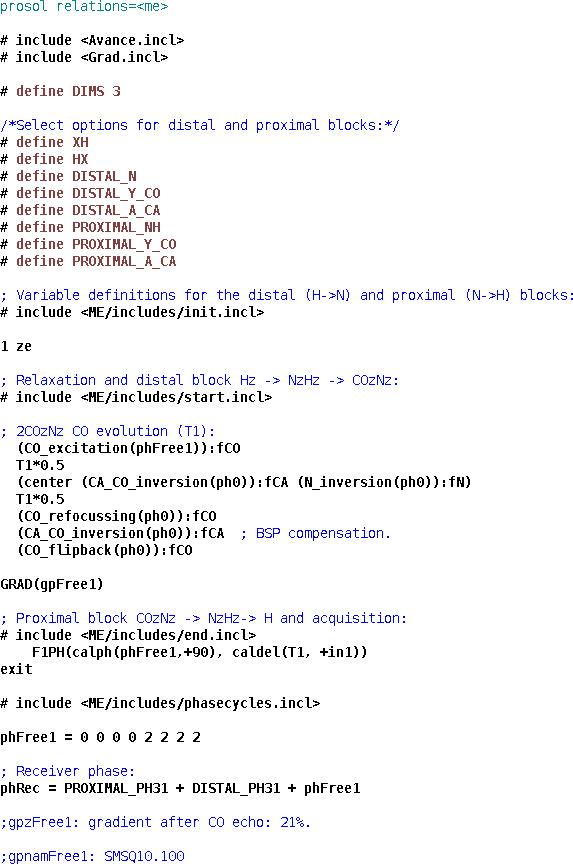
Pulse program code for the implementation of the HNCO experiment.

**Figure 2:**
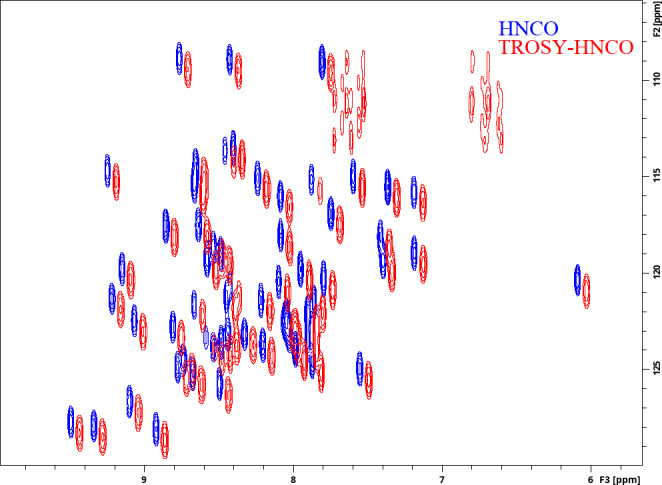
Experimental demonstration of the implementations of the HNCO and TROSY-HNCO experiments for ubiquitin 8 (kDa) at 25 °C. Spectra were recorded as ${}^{1}$ H– ${}^{15}$ N planes with maximum evolution times of 85.2 ms ( ${}^{1}$ H) and 9.87 ms ( ${}^{15}$ N) and processed using cosine-squared window functions.

**Figure 3:**
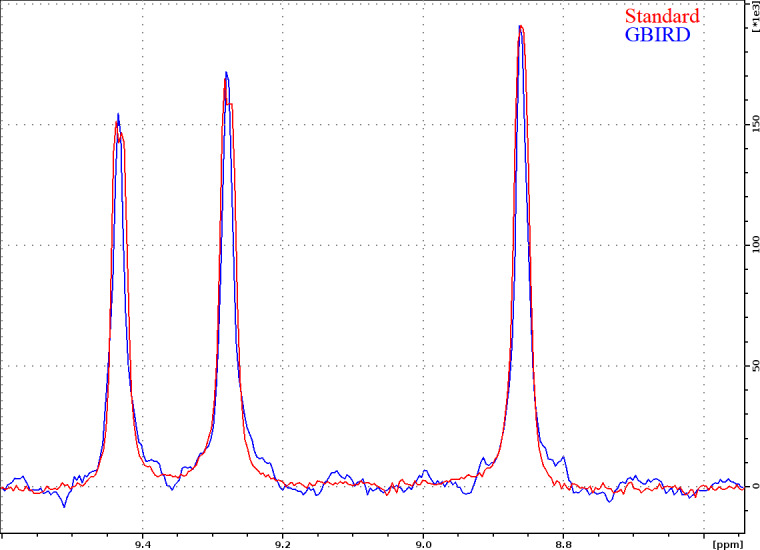
One-dimensional slices (for N=128.5 ppm) through ${}^{1}$ H– ${}^{15}$ N planes recorded for TROSY-HNCO with standard acquisition and TROSY-HNCO with ${}^{13}$ C-GBIRD ${}^{r,X}$ , demonstrating the effectiveness of the homodecoupling and the resultant line narrowing. Both spectra were acquired for ubiquitin 8 (kDa) at 25 °C with maximum evolution times of 340.7 ms ( ${}^{1}$ H) and 9.87 ms ( ${}^{15}$ N), processed using a cosine-squared window function in the N dimension and sine-squared-shifted by $\frac{\pi }{2}$ in the H dimension. The GBIRD spectrum was shifted right by 4 Hz (a shift was possibly induced by sample heating) and scaled up to match the amplitude of the standard TROSY-HNCO. For the GBIRD spectrum, 18 chunks were acquired with a 11.96 ms inter-chunk delay, a 3.5 ms ${}^{2}J_{\mathrm{HC}}$ evolution time and a 120 µ s BIP-720-100-10 (Smith et al., 2001) pulse for ${}^{13}$ C inversion. Line widths at half height are (from left to right) 19.6, 19.5 and 19.1 Hz for the standard spectrum and 13.2, 13.2 and 13.7 Hz for the homodecoupled spectrum (TopSpin peak function).

HNCO is one of the simplest triple-resonance experiments and thus a good candidate for demonstrating the strengths and limitations of the presented approach to library building. We present its ME NMR implementation in Fig. 1. We use a custom prosol file (used mostly for automatic precalculation of pulse parameters) to free up a number of variables. Evolution delays and increments are defined explicitly due to the proximal module's numbered variables (here *td2* and *in2*) being dimensionality-dependent. The block of definitions specifies options for the ME library – the proximal (xh.incl) and distal (hx.incl) modules and the couplings to be evolved ( Y is ${}^{2}J_{\mathrm{NCO}}$ ) and decoupled ( A is ${}^{2}J_{\mathrm{NCA}}$ ). After importing the first two general modules, which include the distal modules and two evolution periods, the carbonyl echo is implemented using the library's low-level functionality. Since channels and pulses are not selected explicitly, this block will function with split CA and CO channels (with the right spectrometer configurations and “CACO_SPLIT” defined in ZGOPTNS) or use a single carbon channel and frequency-offset pulses. The rest of the pulse program includes the end.incl module (with the two proximal echoes and the acquisition) and standard configuration of gradients and phase cycles. To demonstrate the library functionality, in Fig. 2 we present 2D spectra (recorded as HN(CO) experiments) of a standard variant of the experiment (no ZGOPTNs) and a TROSY-HNCO (adding the TROSY definition to ZGOPTNS), selecting only the H ${}_{\beta }$ and N ${}_{\beta }$ components (the lower-right component uses a standard display convention). It is possible to choose a ${}^{13}$ C-GBIRD ${}^{r,X}$ by appending the “ACQ_BIRD_C” option to ZGOPTNS, with an example of line narrowing demonstrated in Fig. 3.

### 4.2 Four-dimensional NOESY

**Figure 4:**
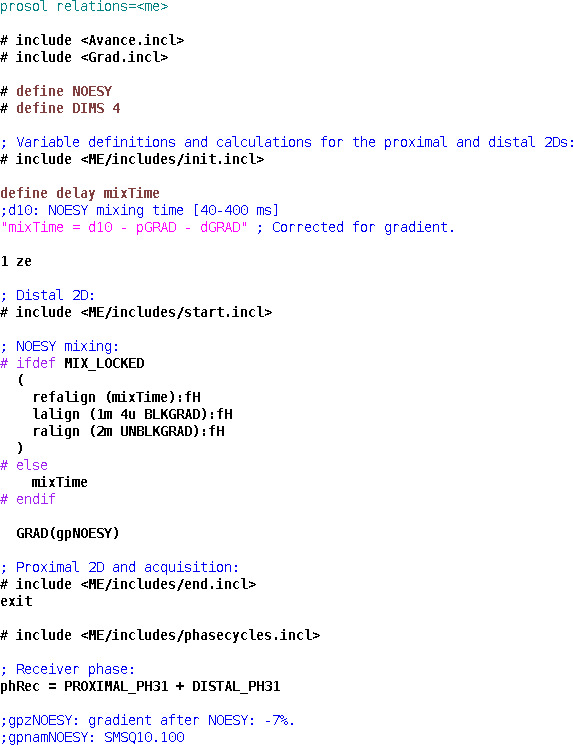
Pulse program code for the implementation of a 4D NOESY experiment.

**Figure 5:**
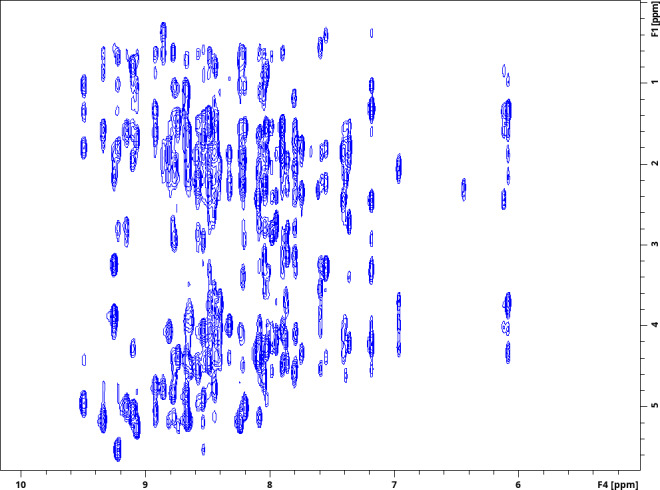
${}^{1}$ H– ${}^{1}$ H planes recorded using a 4D HC,NH-HMQC-NOESY-HSQC experiment for ubiquitin 8 (kDa) at 25 °C. Spectra were recorded with maximum evolution times of 85.2 ms ( ${}^{1}$ H direct dimension) and 6.99 ms ( ${}^{1}$ H indirect dimension) and processed using cosine-squared window functions.

**Figure 6:**
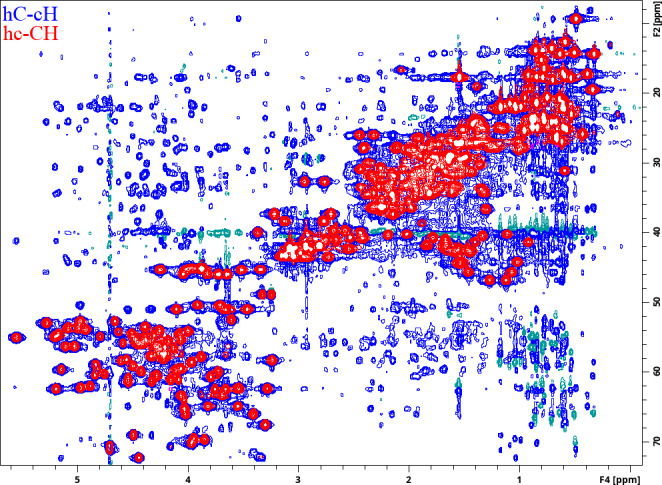
Two different ${}^{1}$ H– ${}^{13}$ C 2D planes recorded using a ME implementation of a 4D HC,CH-HMQC-NOESY-HSQC experiment for ubiquitin 8 (kDa) at 25 °C. Spectra were recorded with maximum evolution times of 85.2 ms ( ${}^{1}$ H direct dimension) and 7.96 ms (both ${}^{13}$ C dimensions) and processed using cosine-squared window functions.

The modular nature of the library is exemplified by the 4D NOESY pulse program in Fig. 4. Apart from the basic structure described above in the case of HNCO, it only contains a mixing period joining the proximal and distal modules, with the evolved heteronuclei and experiment types selected by the user with ZGOPTNS. HC,NH-HMQC-NOESY-HSQC with sensitivity enhancement in the last dimension (Fig. 5) can be changed to a HC,CH-HMQC-NOESY-HSQC (Fig. 6) pulse program by changing the “PROXIMAL_N” option to “PROXIMAL_C” and adding the gradient selection option (“GS”), which is not a default for non-sensitivity-enhanced HSQC.

### 4.3 ${}^{1}$ H– ${}^{15}$ N correlation – shaped pulses

**Figure 7:**
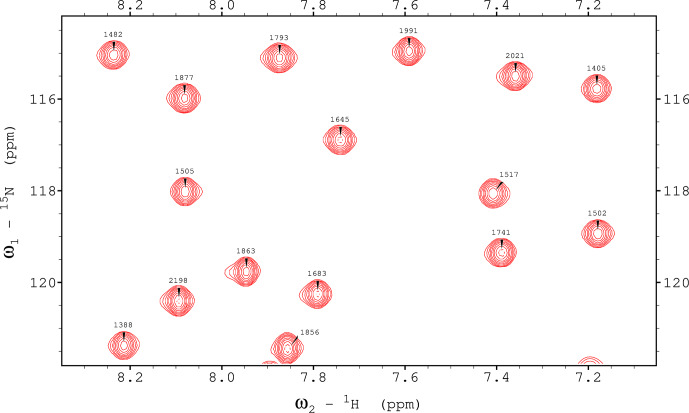
${}^{1}$ H, ${}^{15}$ N TROSY spectrum recorded using a ME implementation with hard pulses and water flipback for ubiquitin 8 (kDa) at 25 °C. The spectrum was recorded with maximum evolution times of 85.2 ms ( ${}^{1}$ H) and 39.5 ms ( ${}^{15}$ N) and processed using cosine-squared window functions.

**Figure 8:**
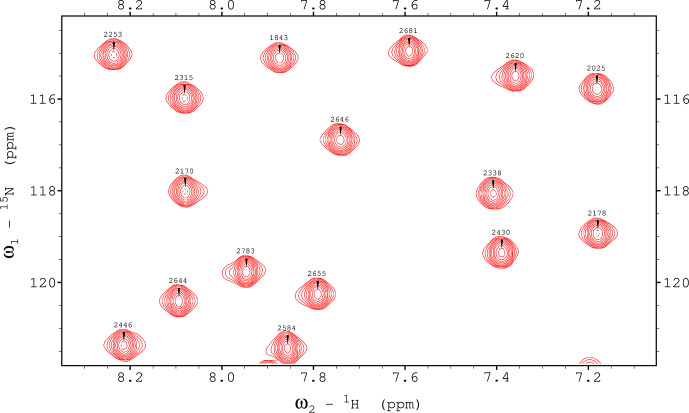
${}^{1}$ H, ${}^{15}$ N TROSY spectrum recorded using a ME implementation with shaped pulses (E400B and RE-BURP) for ubiquitin 8 (kDa) at 25 °C. The spectrum was recorded with maximum evolution times of 85.2 ms ( ${}^{1}$ H) and 39.5 ms ( ${}^{15}$ N) and processed using cosine-squared window functions.

Since BEST-type experiments utilizing shaped pulses can bring improved sensitivity, especially at higher scan repetition rates (Schanda et al., 2006), we demonstrate the library's inherent ability to automatically adapt to the substantial chemical shift and coupling evolution during the 90° universal-rotation E400B (Veshtort and Griffin, 2004) (using a time-reversed version of the original pulse for excitation) pulses with a length of 1073.1 µ s (equivalent to an ideal pulse followed by a 611.7 µ s delay) and refocusing pulse RE-BURP (Geen and Freeman, 1991) with a length of 1108.8 µ s (modelled as an ideal refocusing pulse flanked by 554 µ s delays) in Figs. 7 and 8. With a relaxation delay of 0.65 s, all peaks in the selected region are over 20 % stronger in the shaped-pulse version. Full datasets for a number of different relaxation delays are provided, as in the Data availability section.

## 5 Materials and methods

For all the experiments, we used a 2 mM ${}^{13}$ C, ${}^{15}$ N double-labelled human ubiquitin (ASLA Biotech) in a 5 mm Shigemi NMR microtube. All the spectra were acquired using a Bruker Avance IIIHD 800 MHz spectrometer with a 5 mm TCI z -gradient cryoprobe. Pulse lengths for 90° hard pulses were 10.47 µ s for ${}^{1}$ H, 12.3 µ s for ${}^{13}$ C and 33.22 µ s for ${}^{15}$ N. Full acquisition and processing parameters are provided in the dataset linked below in the Data availability section. Acquisition and library testing were performed using the TopSpin 3.6.5 Service Pack 2 software (Bruker). Data processing and plotting (aside from Figs. 7 and 8) were carried out in TopSpin. Figures 7 and 8 were prepared using the NMRFAM-SPARKY software (Goddard and Kneller, 2004; Lee et al., 2015).

## 6 Conclusions

We have described a framework library implementing a two-level approach to pulse program modularisation and demonstrated its utility. We hope it can be used by others either directly for the streamlining of pulse program code or as an inspiration for similar frameworks. Although the usefulness of the modularisation approach is most obvious for the case of the protein experiments presented here, it should extend to nucleic acids and, to a more limited extent, small molecules. In the latter case, the ability to modularise preparation period operations (presaturation, ASAP), WATERGATE-type (Piotto et al., 1992; Sklenar et al., 1993) solvent suppression and real-time acquisition should be particularly useful.

## Supplement

10.5194/mr-5-51-2024-supplement The supplement related to this article is available online at: https://doi.org/10.5194/mr-5-51-2024-supplement.

## Data Availability

All data used in the preparation of this article are available online at https://doi.org/10.5281/zenodo.10578330 (Górka and Koźmiński, 2024b).

## Code availability

The initial version of the ME library code and documentation is available online at https://doi.org/10.5281/zenodo.10841749 (Górka and Koźmiński, 2024a). The current library version is available online at https://github.com/nmr-cnbch/MEnmr_pubcode (last access: 20 March 2024) or from the authors upon request.

## Appendix

The supplement related to this article is available online at: https://doi.org/10.5194/mr-5-51-2024-supplement.
