# Supplementary material for: A modular library for fast prototyping of solution-state nuclear magnetic resonance experiments
Source: Magn Reson (Gott). 2024 Apr 23;5(1):51–9. doi: 10.5194/mr-5-51-2024 (PMC12590411; doi:10.5194/mr-5-51-2024)
Supplement: The supplement related to this article is available online at: https://doi.org/10.5194/mr-5-51-2024-supplement. [file mr-5-51-2024-supplement.pdf]

Supplement of Magn. Reson., 5, 51–59, 2024
https://doi.org/10.5194/mr-5-51-2024-supplement
© Author(s) 2024. CC BY 4.0 License.

Supplement of

A modular library for fast prototyping of solution-state nuclear magnetic resonance experiments

Michał Górka and Wiktor Koźmiński

Correspondence to: Wiktor Koźmiński (kozmin@chem.uw.edu.pl)

The copyright of individual parts of the supplement might differ from the article licence.

1 Implementation of a HSQC experiment

```
prosol relations=<me>

# include <Avance.incl>
# include <Grad.incl>

# define DIMS 2

; Variable definitions for the 2D block:
# include <ME/includes/init.incl>

1 ze

; Relaxation delay:
# include <ME/includes/start.incl>

; 2D (proximal) block:
# include <ME/includes/end.incl>
exit

# include <ME/includes/phasecycles.incl>

; Receiver phase:
phRec = PROXIMAL_PH31
```

Fig. S1. Pulse program code for the ME implementation of a 2D experiment.

We will illustrate the functioning of the library using a (non-sensitivity enhanced) 2D HSQC experiment. This experiment is set up by using the 2D.me.pp pulse program of Fig. S1. and selecting the HSQC option by appending –DHSQC to *ZGOPTNS*. A ^{1}H-^{13}C experiment (^{1}H-^{15}N is the default) can be selected by appending –DPROXIMAL_C to *ZGOPTNS*.

The program uses a custom prosol file and imports the standard TopSpin library files Avance.incl and Grad.incl containing macros for controlling the lock system and gradient amplifier. After defining the dimensionality of the experiment as 2 the init.incl file performs setup of the ME library and includes the chosen 2D module for the first time. The ze statement opens the real-time portion of the pulse program and the following start.incl file implements the relaxation delay with optional solvent presaturation (-DPRESAT in *ZGOPTNS*). It includes the chosen 2D module (here it is hsqc.me.pp) for the second time, but in the case of a 2D HSQC this has no effect. The end.incl module includes the 2D HSQC module for a third time implementing the three corresponding evolution periods of the experiment. Afterwards the end.incl module includes an acquisition block (with decoupling and options for homodecoupling). The exit statement ends the real-time part of the experiment and the following phasecycles.incl file sets up a 4 step phase cycle. The receiver phase is calculated using the PROXIMAL_PH31 macro defined in the HSQC module – in more complex experiments user-defined phase cycles can be added to the macro.

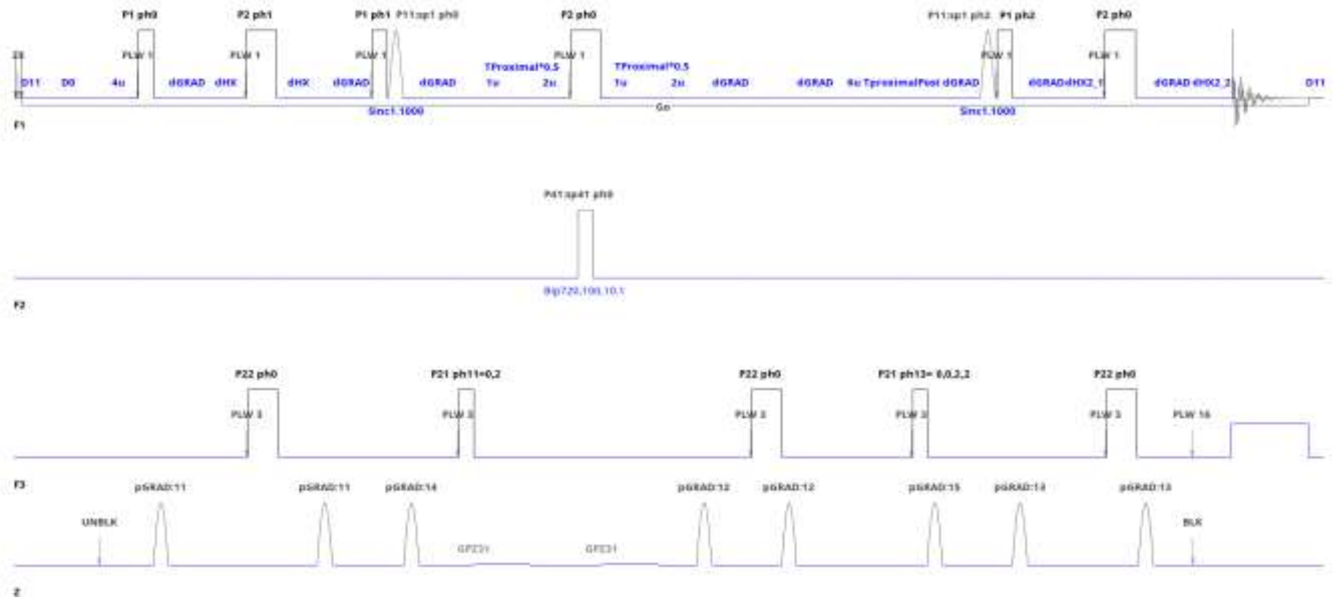

Fig. S2. Pictorial representation of a HSQC experiment implemented using the ME library.

A graphical representation of a 2D HSQC ^{1}H-^{13}C with radiation dampening gradients (-DRDGRAD in *ZGOPTNS*), water flipback (-DFB in *ZGOPTNS*) and gradient selection (-DGS in *ZGOPTNS*) implemented using the pulse program from Fig. S1. is presented in Fig. S2. The full version of the hsqc.me.pp program is available in the library, here we will analyze each of its sections separately.

```
1  # ifndef INIT_DONE
2
3  ; Set up chemical shift evolution time:
4  define delay TProximal
5  "TProximal = 0"
6
7  define delay TproximalPost  ;
8  define delay dHX  ; H -> HXz period.
9  define delay dHX2_1  ; Second H period - before acquisition.
10 define delay dHX2_2  ; Second H period - before acquisition.
11
12 ; Compensate J evolution delays for gradients and evolution during pulses:
13 "dHX = timeHX/2 - pGProximalL - dGProximalL - eHX_excitation"
14 "dHX2_1 = timeHX/2 - pGRAD - dGRAD - eHX_WATERGATE/2 - eHX_excitation"
15 "dHX2_2 = timeHX/2 - pGRAD - dGRAD - eHX_WATERGATE/2 - ME_de"
16
17 ; Compensate for inversion pulses during X SQ evolution:
18   "TproximalPost = larger(pHX_inversion, pY_inversion)"
19
20 /*For FB - determine whether solvent should be +z or -z after last H excitation pulse:*/
21 # if defined(WG)
22 /*  WATERGATE pulse won't invert solvent -> solvent to z:*/
23 #   define phPROXIMAL_SOLVENT ph2
24 # else
25 /*  If there is inversion in the second INEPT leave solvent along -z after first INEPT:*/
26 #   define phPROXIMAL_SOLVENT ph0
27 # endif
28
29 ; Phase of last H excitation pulse:
30 # ifdef GS
31 #   define phGS ph3
32 # else
33 #   define phGS ph2
34 # endif
35
36 "acqt0 = 0"
37 baseopt_echo
38 ; *****************************************************************************
39 # elif defined(INIT_DONE) && !defined(ME_PULSES)
40
41 ; *****************************************************************************
42 # elif defined(ME_PULSES) && !defined(IMPORT_PHASES)
43
```

Fig. S3. The first part of the hsqc.me.pp file implementing the proximal HSQC module.

First section (at the init.incl position) of the code is presented in Fig. S3. and contains the code for the calculation of lengths of delays. After defining the needed variables the lengths of the delays for the evolution of $^{1}J_{HX}$ scalar coupling are adjusted to accommodate optional gradients (*pGRAD*), gradient delays (*dGRAD*) and the effective evolution during pulses flanking pulses (*eHX_excitation)* and (if selected in *ZGOPTNS*) the WATERGATE block (*eHX_WATERGATE*). The *TproximalPost* delay is set to compensate for the length of the inversion pulses (for H and optionally Y – without Y nuclei pY_inversion is set to 0 s elsewhere) in the middle of the X chemical shift evolution period.

The part of the module that is included in a pulse program at the position of the start.incl file (line 40) is empty, as no real-time calculations need to be performed for a standard HSQC.

```
; ******************************************************************************
# elif defined(ME_PULSES) && !defined(IMPORT_PHASES)


# ifndef PROXIMAL_X_START
; Hz -> HzXz:
  (HX_excitation(ph0)):fH
  PROXIMAL_GRAD_LONG(gpProximal1)
  dHX
  (center (HX_refocussing(ph1)):fH (X_inversion(ph0)):fX)
  dHX
  PROXIMAL_GRAD_LONG(gpProximal1)
  (HX_flipback(ph1)):fH
  H2O_FLIPBACK(ph0)
# endif /*X_START*/

  PROXIMAL_GRAD_LONG(gpProximal4)

; HzNz -> HzNy + HzNx with N evolution (T1):
  (X_excitation(phProximal1)):fX
  RDGRAD_ON
  TProximal*0.5
  RDGRAD_OFF
  (center (HX_inversion(ph0)):fH (Y_inversion(ph0)):fY)
  RDGRAD_ON_NEG
  TProximal*0.5
  RDGRAD_OFF
  GRAD_PROXIMAL(gpProximalX,gpProximal2)
  (X_refocussing(ph0)):fX
  GRAD_PROXIMAL(gpProximalX2,gpProximal2)
  RDGRAD_COMP
  TproximalPost
  (X_flipback(phProximal3)):fX

  PROXIMAL_GRAD_LONG(gpProximal5)

; HzXz -> Ht:
  H2O_FLIPBACK(phGS + phPROXIMAL_SOLVENT)
  (HX_excitation(phGS)):fH
  GRAD_PROXIMAL_GS(gpProximalH,gpProximal3)
  dHX2_1
  (center (HX_WATERGATE(ph0)):fH (X_refocussing(ph0)):fX)
  GRAD_PROXIMAL_GS(gpProximalH2,gpProximal3)
  dHX2_2 CPD_POW BLKGRAD

# ifndef GS
#   define PROXIMAL_MC1 F1PH(calph(phProximal3, +90), caldel(TProximal, +in1))
#   define PROXIMAL_MC2 F2PH(calph(phProximal3, +90), caldel(TProximal, +in2))
#   define PROXIMAL_MC3 F3PH(calph(phProximal3, +90), caldel(TProximal, +in3))
#   define PROXIMAL_MC4 F4PH(calph(phProximal3, +90), caldel(TProximal, +in4))
# else
#   define PROXIMAL_MC1 F1EA(calgrad(EA), caldel(TProximal, +in1) & calph(phProximal1, +180) & calph(phRec, +180))
#   define PROXIMAL_MC2 F2EA(calgrad(EA), caldel(TProximal, +in2) & calph(phProximal1, +180) & calph(phRec, +180))
#   define PROXIMAL_MC3 F3EA(calgrad(EA), caldel(TProximal, +in3) & calph(phProximal1, +180) & calph(phRec, +180))
#   define PROXIMAL_MC4 F4EA(calgrad(EA), caldel(TProximal, +in4) & calph(phProximal1, +180) & calph(phRec, +180))
# endif

; ******************************************************************************
```

Fig. S4. The second part of the hsqc.me.pp file implementing the proximal HSQC module.

Fig. S4. presents the third section of the code (at the end.incl position) implementing the actual HSQC experiment. All pulses are implemented using the libraries low-level functionality (with a HX_ prefix for protons and X_ prefix for the active heteronucleus) and can thus be switched between hard and shaped pulses and the length of delays (such as *dHX*) will be automatically adjusted as per the previous section – for *dHX* using *eHX_excitation*.

The first evolution period (lines 57-64) implements an INEPT transfer to the X nucleus with optional gradients flanking the H refocusing pulse. The optional flipback pulse H2O_FLIPBACK will be skipped if the options –DFB is not selected using ZGOPTNS. The initial block can be skipped by setting the macro PROXIMAL_X_START – the experiment than use X steady steady-state magnetization instead of protons.

The second evolution period (lines 70-83) implements an X chemical shift evolution period with refocusing of the coupling to protons and optionally to Y nuclei. Gradients can be used to mitigate radiation dampening for longer maximum evolution times in the X dimension. In this case RD_GRAD (RD_GRAD_NEG) will be replaced by a statement switching on a positive (negative) square gradient with RDGRAD_OFF switching it off. If the RDGRAD option is not used all those macros will have no effect. The GRAD_PROXIMAL macro enable the implementation of three different options. If gradient selection is desired (-DGS in *ZGOPTNS*) gradients on two sides of the X refocusing pulse will have different amplitudes. With extra gradients (-DGRAD_SHORT) the two gradients will have equal amplitudes and without any of the two options the statements will have no effect.

The third evolution period (lines 88-94) implements the second INEPT block. Solvent flipback is implemented as in the first INEPT and selection gradients as in the second evolution period. Instead of a standard HX_refocussing pulse a HX_WATERGATE variant is used. Without the appropriate option (-DWG in ZGOPTNS) it will have the same effect as HX_refocussing, but with the option turned on a selected WATERGATE-type block will be used – by default it is W5 with corrections (Liu et al., 1998).

The rest of this section (lines 96-106) illustrates one of the limitations of the pulse programming language used. Since this module can also be used in a 3 or 4D NOESY multiple variants of an MC statement are specified. For gradient selected experiments echo-antiecho MC statements are used and “standard” (TPPI/States-TPPI/States) otherwise.

```
109 # elif defined(IMPORT_PHASES)
110
111 ; X excitation:
112 # if !defined(DISTAL_PHASES_2)
113     phProximal1 = 0 2
114 #   define PROXIMAL_PHASES_2
115 # elif !defined(DISTAL_PHASES_4)
116     phProximal1 = 0 0 2 2
117 #   define PROXIMAL_PHASES_4
118 # else
119     phProximal1 = 0 0 0 0 2 2 2 2
120 #   define PROXIMAL_PHASES_8
121 # endif
122
123 ; X flipback:
124 # if !defined(DISTAL_PHASES_4) && !defined(PROXIMAL_PHASES_4)
125     phProximal3 = 0 0 2 2
126 # elif !defined(DISTAL_PHASES_8) && ! defined(PROXIMAL_PHASES_8)
127     phProximal3 = 0 0 0 0 2 2 2 2
128 # elif !defined(DISTAL_PHASES_16) && ! defined(PROXIMAL_PHASES_16)
129     phProximal3 = 0 0 0 0 0 0 0 0 2 2 2 2 2 2 2 2
130 # else
131     phProximal3 = 0
132 # endif
133
134 # define PROXIMAL_PH31 phProximal1 + phProximal3
135
136 ;gpzProximal1: Gradient in Hz -> HzXz echo: 1%.
137 ;gpzProximal2: Short gradient in X evolution echo: 5%.
138 ;gpzProximal3: Short gradient in HzXz -> Ht (WATERGATE) echo: 21%.
139 ;gpzProximal4: Gradient after Hz -> HzXz echo: 3%.
140 ;gpzProximal5: Gradient after X evolution echo : 11%.
141
142 ;gpzProximalX: First coherence selection gradient (on N/C): 80%.
143 ;gpzProximalX2: Second coherence selection gradient (on N/C): 0%.
144 # ifndef PROXIMAL_C
145 ;gpzProximalH: Third coherence selection gradient (on H): 1%.
146 ;gpzProximalH2: Forth coherence selection gradient (on H): 9.1%.
147 # else
148 ;gpzProximalH: Third coherence selection gradient (on H): 1%.
149 ;gpzProximalH2: Forth coherence selection gradient (on H): 21.1%.
150 # endif
151
152 # endif /*INIT DONE*/
```

Fig. S5. The first part of the hsqc.me.pp file implementing the proximal HSQC module.

The third section (Fig. S5.) defines phase programs and contains gradient amplitude suggestions (lines 136-152). Since in NOESY experiments the distal module will be included before this proximal module it will signal which lengths of phase cycles it uses by setting the corresponding macros – by default it will be DISTAL_PHASES_2 and DISTAL_PHASES_8. The HSQC module will therefore use a {x, -x} cycle for the X excitation pulse in a 2D experiment and a {x, x, -x, -x} cycle in a NOESY experiment. The macro PROXIMAL_PH31 is set appropriately to complement the phase cycles of pulses.

2 Example configuration utility for 4D NOESY

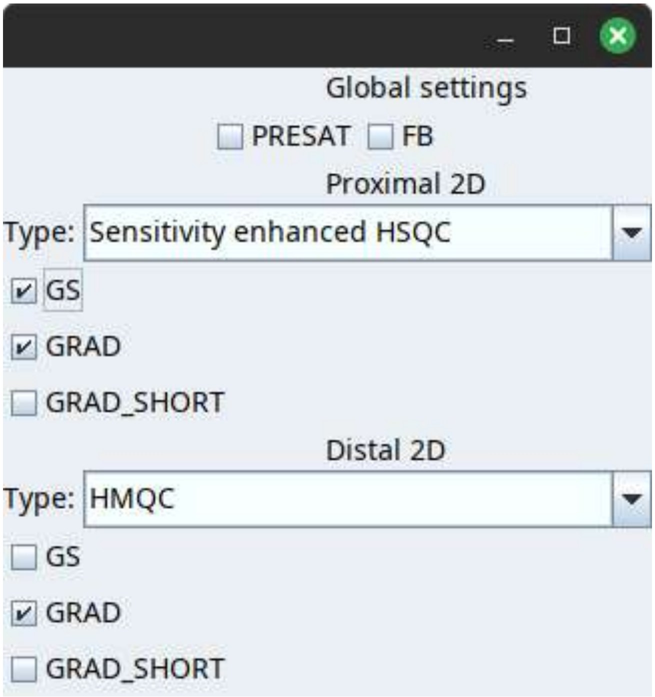

Fig. S6. me.config.py GUI configurator for the 4D NOESY pulse program.

In Fig. S6. we present the GUI of an experimental configuration utility "me.config.py" for the 4D NOESY pulse program. The *ZGOPTNS* variable is updated after each selection. The utility is experimental, since the underlying TopSpin Jython infrastructure is underdocumented and the utility suffers some performance problems. Changing the active TopSpin window during program operation can also lead to bugs or unintentional changes to other experiments *ZGOPTNS* variable. Nonetheless we think that the source code of the program can serve as an inspiration or be re-used in other efforts in building a pulse program library – whether it is based on ME or not.

References

Liu, M., Mao, X., Ye, C., Huang, H., Nicholson, J. K., and Lindon, J. C.: Improved WATERGATE Pulse Sequences for Solvent Suppression in NMR Spectroscopy, Journal of Magnetic Resonance, 132, 125–129, https://doi.org/10.1006/jmre.1998.1405, 1998.
